# Supplementary figures and images for: A comparison of chloroplast genome sequences in Aconitum (Ranunculaceae): a traditional herbal medicinal genus
Source: PeerJ. 2017 Nov 7;5:e4018. doi: 10.7717/peerj.4018 (PMC5680694; doi:10.7717/peerj.4018)

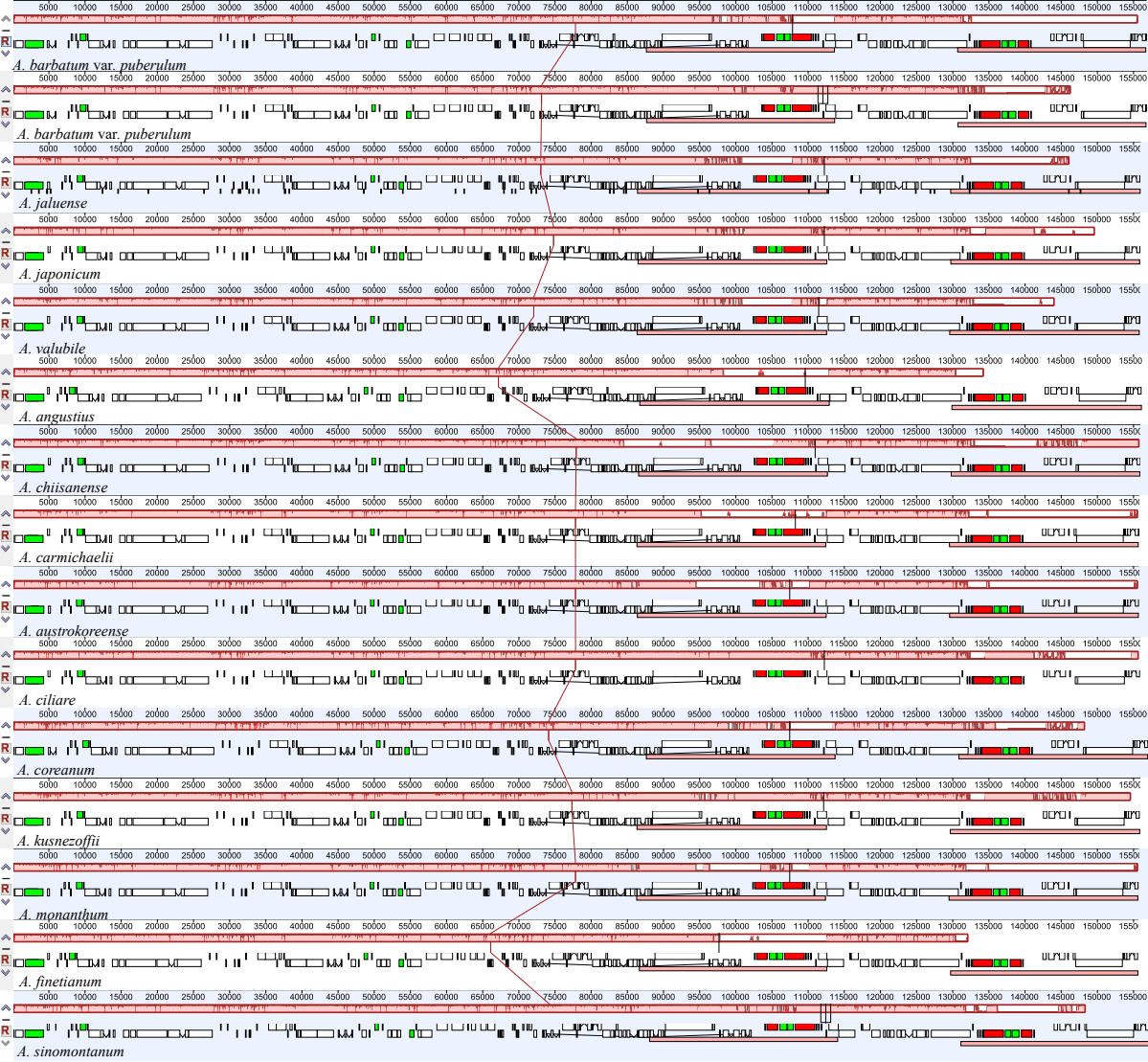

Supplement: Figure S1 — The boxes above and below the line represent the gene sequences in clockwise and anticlockwise directions, respectively. The gene names at the bottom indicate the genes located at the boundaries of the boxes in cp genome of Aconitum. [file peerj-05-4018-s001.pdf]
