# Supplementary material for: A comparison of chloroplast genome sequences in Aconitum (Ranunculaceae): a traditional herbal medicinal genus
Source: PeerJ. 2017 Nov 7;5:e4018. doi: 10.7717/peerj.4018 (PMC5680694; doi:10.7717/peerj.4018)
Supplement: Table S1 [file peerj-05-4018-s002.docx]

**Table S1 Gene contained in the sequenced chloroplast genomes of three *Aconitum* species.**

| **Category** | **Gene group** | **Gene name** | | | | |
| --- | --- | --- | --- | --- | --- | --- |
| Self-replication | Ribosomal RNA genes | *rrn16*^a^ | *rrn23*^a^ | *rrn4.5*^a^ | *rrn5*^a^ |  |
|  | Transfer RNA genes | *trnA-UGC^a,,b^* | *trnC-GCA* | *trnD-GUC* | *trnE-UUC* | *trnF-GAA* |
|  |  | *trnfM-CAU* | *trnG-GCC^b^* | *trnH-GUG* | *trnI-CAU^a^* | *trnI-GAU^a,b^* |
|  |  | *trnK-UUU^b^* | *trnL-CAA^a^* | *trnL-UAA^b^* | *trnL-UAG* | *trnM-CAU* |
|  |  | *trnN-GUU^a^* | *trnP-UGG* | *trnQ-UUG* | *trnR-ACG^a^* | *trnR-UCU* |
|  |  | *trnS-GCU* | *trnS-GGA* | *trnS-UGA* | *trnT-GGU* | *trnT-UGU* |
|  |  | *trnV-GAC^a^* | *trnW-CCA* | *trnY-GUA* | *trnG-UCC* | *trnP-GGG* |
|  |  | *trnV-UAC* |  |  |  |  |
|  | Small subunit of ribosome | *rps11* | *rps12^c,d^* | *rps14* | *rps15* | *rps18* |
|  |  | *rps19* | *rps2* | *rps3* | *rps4* | *rps7^a^* |
|  |  | *rps8* | *rps16* |  |  |  |
|  | Large subunit of ribosome | *rpl14* | *rpl16^b^* | *rpl2^b^* | *rpl20* | *rpl22* |
|  |  | *rpl23^a^* | *rpl33* | *rpl36* |  |  |
|  | DNA-dependent RNA polymerase | *rpoA* | *rpoB* | *rpoC1^b^* | *rpoC2* |  |
| Genes for photosynthesis | Subunits of photosystem I | *psaA* | *psaB* | *psaC* | *psaI* | *psaJ* |
|  |  | *ycf1* | *ycf3^c^* | *ycf4* | *ycf15* |  |
|  | Subunits of photosystem II | *psbA* | *psbB* | *psbC* | *psbD* | *psbE* |
|  |  | *psbF* | *psbH* | *psbI* | *psbJ* | *psbK* |
|  |  | *psbL* | *psbM* | *psbN* | *psbT* | *psbZ* |
|  | Subunits of cytochrome | *petA* | *petB^b^* | *petD^b^* | *petG* | *petL* |
|  |  | *petN* |  |  |  |  |
|  | Subunits of ATP synthase | *atpA* | *atpB* | *atpE* | *atpF^b^* | *atpH* |
|  |  | *atpI* |  |  |  |  |
|  | Large subunit of Rubisco | *rbcL* |  |  |  |  |
|  | Subunits of NADH dehydrogenase | *ndhA^b^* | *ndhB^a,b^* | *ndhC* | *ndhD* | *ndhE* |
|  |  | *ndhF* | *ndhG* | *ndhH* | *ndhI* | *ndhJ* |
|  |  | *ndhK* |  |  |  |  |
| Other genes | Maturase | *matK* |  |  |  |  |
|  | Envelope membrane protein | *cemA* |  |  |  |  |
|  | Subunit of acetyl-CoA | *accD* |  |  |  |  |
|  | C-type cytochrome synthesis gene | *ccsA* |  |  |  |  |
|  | Protease | *clpP^c^* |  |  |  |  |
|  | Function unknown | *ycf2^a^* |  |  |  |  |
|  | Translation initial factor | *infA* |  |  |  |  |

a: Two gene copies in IRs; b: gene containing an interval; c: gene containing two intervals; d: gene divided into two independent transcription units.
